# Multicentric pragmatic randomized controlled trial of mesh fixation system in primary and recurrent ventral hernia repair

**DOI:** 10.1007/s13304-026-02817-1

**Published:** 2026-09-07

**Authors:** Francesco Prete, Giuseppe Massimiliano De Luca, Francesco Vittore, Alessandro Pasculli, Luigi Marano, Giuseppe Carbotta, Giuseppe Cavallaro, Diego Cuccurullo, Lorenzo Fracasso, Stefania Romano, Giorgio Soliani, Carlo Sagnelli, Mariasole Fracasso, Riccardo Maglio, Silvia Malerba, Giuliana Rachele Puglisi, Alfio Gianalberto Testini, Michele Covelli, Angela Gurrado, Giampiero Campanelli, Mario Testini

**Affiliations:** 1 Academic General Surgery Unit “F. Paccione”, Department of Precision and Regenerative Medicine and Jonian Area (DiMePRe-J), University of Bari Aldo Moro Medical School, Bari (BA), Italy; 2 Department of Surgery, Academy of Applied Medical and Social Sciences - AMiSNS, Elblag, Poland; 3 General Surgery Unit, S. Giuseppe Di Copertino Hospital, Copertino, Lecce (LE), Italy; 4 Department of Surgery, Sapienza University, Rome, Italy; 5 Laparoscopic and Robotic Surgery Unit, Monaldi Hospital, Naples, Italy; 6 General Surgery Unit, Scorrano Hospital Centre, Scorrano, Lecce (LE), Italy; 7 General Surgery Unit, Casarano Hospital Centre, Casarano, Lecce (LE), Italy; 8 General and Abdominal Wall Surgery Unit, “Ospedale SS Annunziata Di Cento”, Ferrara, Italy; 9 Department of Medicine, LUM University, Casamassima, Bari (BA), Italy; 10 General Surgery, Day and Week Surgery Unit, Galeazzi – Sant’Ambrogio IRCCS Hospital, Insubria University, Varese, Italy

**Keywords:** Abdominal wall hernia, Incisional Hernia, Ventral Hernia, Mesh fixation, Cyanoacrylate, Glubran II, Glutack, Randomized controlled trial

## Abstract

Controversy persists regarding the ideal type of fixation for hernia mesh. Transfixing methods have been linked to tissue trauma and to the development of chronic pain. In ventral hernia repair (VHR), retrospective studies showed a potential for fixation with cyanoacrylate of being as effective as conventional suture or tacks methods but with less postoperative pain and complications. A pragmatic randomized trial was conducted on patients undergoing primary and incisional VHR, analyzing mesh fixation by cyanoacrylate (experimental group) versus sutures or reabsorbable tacks (control group), stratifying a priori by laparoscopic(LVHR) and open approach(OVHR) to account for distinct anatomical and biomechanical environments. Primary outcome was chronic postoperative pain. Secondary outcomes were postoperative complications, recurrence, operating time, intraoperative blood loss, pain during admission. Univariable and multivariable analysis were conducted. Two hundred fourteen patients were included, of which 109 treated laparoscopically. Overall chronic pain occurred exclusively within the open surgery cohort (12/105 cases, 11.4%), with no statistical difference between fixation arms. Cyanoacrylate for mesh fixation was independent factor for fewer perioperative complications (OR 0.457, 95% CI 0.244–0.855, *p* = 0.014), shorter operating time (− 7.172 min, 95%CI − 23.040/− 2.008, *p* = 0.02), reduced pain at hospital discharge (− 0.356 VAS point, 95%CI − 0.691/− 0.022, *p* = 0.037). In LVHR, non-transfixing mesh positioning was also a factor for less intraoperative blood loss. In OVHR there were no significant difference in outcomes between groups. In a pragmatic real-world setting, long-term chronic pain appears primarily driven by surgical access and wound-related factors rather than the independent effect of the mesh fixation method. Cyanoacrylate fixation provides significant perioperative advantages, particularly in LVHR.

## Introduction

Anterolateral abdominal wall defects are a common surgical presentation and indication for surgery worldwide, with an estimated prevalence of about 5% in the general population [1]. It has been estimated that about three quarters of ventral hernia defects are primary in etiology, predominantly epigastric and umbilical, with the remaining 25% occurring as incisional defects [2]. Incisional hernia remains a prevalent complication after open abdominal surgery, ranging from 2 to 20%, and can be as high as 69% in cohorts of high-risk patients [3–5].

The current standard of care consists of abdominal wall repair with mesh, utilizing either an open or minimally invasive approach [6]. The optimal fixation method for prosthetic material remains a subject of debate, particularly during laparoscopic repair, as controversy persists regarding the ideal strength, quantity, and type of fixation material required, whether absorbable or permanent [7–9].

Historically, sutures or tacks have been the two main categories of fixation methods available for use in the operating room, both of which are available in absorbable or permanent varieties [9]. Although these mechanical methods provide necessary biomechanical stability, they have been associated with potentially severe complications due to their perforating nature [10–12]. Specifically, spiral tacks have been linked to nerve entrapment resulting in postoperative pain, intestinal obstruction, and, in rare instances, fistulae or small bowel perforation [11, 13, 14].

One of the most frequent complications after abdominal wall hernia surgery is post-operative pain, which may persist more than six months and become chronic. Sutures, anchors, tacks, and staples have all been linked to tissue trauma and to the development of chronic pain [15–17].

To reduce perforation-associated complications and chronic pain, tissue sealants have been proposed as an alternative to mechanical fixation devices, offering mesh stabilization avoiding tissue penetration [18, 19]. While fibrin and cyanoacrylate-based adhesives are currently established in hernia surgery, especially in inguinal hernia repair, their widespread surgical adoption remains limited [20–23].

Regarding fixation during ventral hernia repair (VHR), laboratory and human reports have utilized surgical adhesive sealants, showing optimal host tissue integration and long-term bio-mechanical resistance of fixation, yet the available evidence is limited [24, 25].

Retrospective studies showed a potential for fixation with cyanoacrylate of being as effective as conventional suture or tacks methods while reducing postoperative pain and complications [26–28]. Real-world herniology deals with a diverse spectrum of defects and access methods; biomechanical, physiological, and wound-healing environments vary between open extraperitoneal repairs (fixation interfaces with dissected fascial planes) and laparoscopic intraperitoneal repairs (where fixation elements may interface directly with visceral structures) [15, 29].

Consequently, while randomized clinical trials often mandate complete procedural uniformity, this study was designed as a pragmatic multi-center trial aiming to assess, under real-world conditions, the safety and effectiveness of cyanoacrylate mesh fixation compared to mechanical transfixion, evaluating laparoscopic and open repairs as independent surgical cohorts to account for access-driven clinical heterogeneity.

## Methods

A pragmatic, multicentre randomized trial was designed and conducted in Italy across seven participating centres certified by the European Hernia Society (EHS) and the Italian Society of Hernia and Abdominal Wall Surgery (ISHAWS). The protocol for this study was prospectively approved by the Institutional Review Board of the University of Bari (UniBA prot. n. 30,594/2023).

The study compared non-transfixing mesh anchoring using synthetic cyanoacrylate-based glue (Glubran® 2; Experimental Group) or mesh transfixing by monofilament sutures or absorbable tacks (Control Group) in ventral hernia repair with mesh.

In line with trial design, surgeons were allowed to use their preferred techniques, choice of hernia mesh and surgical approach, open or minimally invasive, within the assigned method. Procedural heterogeneity was intentionally permitted to maximize external validity and reflect contemporary abdominal wall practice across certified hernia centers.

To ensure basic procedural standardization across the seven participating certified centers, all participating operating surgeons were required to be senior board-certified general surgeons with verified expertise in advanced abdominal wall reconstruction and active registry membership in the EHS or ISHAWS.

The primary aim was to investigate differences between the two groups regarding postoperative chronic pain as the primary endpoint, alongside several secondary endpoints.

### Non-transfixing and transfixing methods of mesh positioning

Cyanoacrylate is the generic name of a group of fast-acting adhesives (such as ethyl-2-cyanoacrylate, *n*-butyl cyanoacrylate, 2-octy1 cyanoacrylate). As a liquid acrylate monomer, cyanoacrylate polymerizes exothermically in the presence of water, especially with hydroxide ions, joining the bonded surfaces in 4–5 s and reaching the final stage in 60–90 s. It bonds body tissue excellently and shows bacteriostatic effects. The film of glue is eliminated by hydrolytic breakdown, a process whose duration varies according to tissue type and quantity.

Glubran® 2, synthetic biodegradable cyanoacrylate-based glue (an *N*-Butyl 2 Cyanoacrylate [NBCA]) modified by the addition of a monomer (Methacryloxysulfolane [MS]). In contrast to most cyanoacrylate glues used only for external applications as skin wound closure, Glubran® 2 is a class III medical device CE-certified for both external and internal use, and previously demonstrated to be well-tolerated and safe [30].

To apply Glubran® 2 in precise drops a dedicated device has been designed in two versions, for laparoscopic procedures and for open surgery. Glutack® is a CE-marked medical device system for glue application in laparoscopic mesh fixation procedures, fabricated to offer a precise and controlled delivery of Glubran® glues.

During open surgical procedures, the Glutack® Short device enables the application of Glubran® 2 in the form of calibrated drops (0.0125 ml/drop–12.5 mg). It is primarily designed for the delivery of Glubran® 2 in the atraumatic fixation of prosthetic meshes during inguinal hernia and incisional hernia repairs. The device ensures the controlled application of the Glubran® 2 surgical sealant via calibrated drops in open surgery settings.

Transfixing mesh fixation could be performed by using either slow-absorbing sutures, monofilament or barbed, or absorbable, self-retaining fastener devices that could be screw-shaped, U-shaped or barbed.

For all laparoscopic techniques, mesh fixation was strictly mandated according to the randomly allocated treatment arm.

### Inclusion criteria and study protocol

Inclusion and exclusion criteria are summarized in Table 1.

**Table 1:** Inclusion and exclusion criteria

*Inclusion criteria*
1. Age > 18 years;
2. Clinical diagnosis of:
(a) primary ventral hernia
(b) primary abdominal border hernia
(c) incisional hernia
(d) incisional hernia and complex abdomen
(e) swiss-cheese defects or multiple small defects, if treatable with only one mesh
3. No significant cardiopulmonary, hepatic, or renal impairment, and no contraindications for surgery;
4. ASA grade I to III;
5. Patient’s consent to treatment (Informed consent signed prior to treatment)
*Exclusion criteria*
1. Incarcerated hernia;
2. Recent allergic disease, or history of atopic allergy;
3. Patients with ASA IV or V;
4. Participations in other clinical studies in the 3 months before surgery;
5. Use of drugs that are known harmful to vital organs during the 4 weeks before surgery;
6. Active infection located at the surgical site or bacteraemia at the time of randomization;
7. Patients with collagenopathy or other relevant concurrent diseases;
8. Patient with neurological disorders and/or mood disorders; history of mental illness;
9. Pregnancy
10. Diseases that may significantly increase Intra-Abdominal Pressure (IAP) and cannot be effectively controlled such as severe ascites, severe asthma caused by bronchitis, pulmonary emphysema, or urine retention caused by significant Benign Prostatic Hyperplasia (BPH)

Patients were referred by their general practitioners or other hospital departments and recruited in the outpatient clinic.

At the baseline clinic visit, an investigator recorded demographic data, medical history, and clinical assessment data, followed by a comprehensive physical examination and evaluation of clinical signs and symptoms. During this visit, patients received both verbal and written information regarding the study before providing written informed consent. All included patients underwent a physical examination and a CT scan of the abdominal wall with a *Valsalva* maneuver prior to surgery to confirm the diagnosis. The final size of the hernia defect was determined intraoperatively.

Pre-study power calculations identified a target sample size of 100 patients per arm. Patients were followed for 6 months, with six postoperative check-ups scheduled according to the study protocol (at 24 h and at 7, 14, 90, and 180 days).

### Random allocation and blinding

Patients were randomly assigned (1:1 ratio) to one of the two treatment groups via a centralized program requiring logged access. The application of the fixation devices was performed by investigators who had received standardized training on the assembly and use of each system before the initiation of the study.

Patients were blinded to the method of fixation used during ventral hernia repair (VHR). Deidentified patient data were entered in a centralized, encrypted database where participating centre, operating surgeon and type of procedure were also codified. Data analysts remained blinded to the experimental conditions.

The postoperative recovery protocol was standardized across all participating Centres and included early mobilization and resumption of oral diet, and a dedicated postoperative pain protocol.

### Postoperative pain control

All participating Centres adhered the same postoperative analgesia protocol, that included paracetamol 1 g every 4–6 h (max 4 tabs/day) and NSAIDs/COX-2 Inhibitors (Ibuprofen, 400 mg or similar non-steroidal anti-inflammatory drugs) for incisional hernias EHS W1-W2. For EHS W3 ventral hernias, a combination of single-shot transversus abdominis plane (TAP) regional anesthesia block with standard ropivacaine or bupivacaine, paracetamol 1 g every 4–6 h (max 4 tabs/day) and NSAIDs/COX-2 Inhibitors.

### Follow up

Follow-up visits occurred at 24 h, one week, two weeks, three months and six months. During these visits, clinical assessments were performed on safety, effectiveness and pain metrics using the revised International Association for the Study of Pain (IASP) definitions [31]. Regarding effectiveness, the mesh implant area was evaluated for recurrence through a focused abdominal examination. In cases of suspected recurrence, an ultrasound or CT/ MRI was performed according to hospital practice guidelines to confirm diagnosis. Long-term tracking at the 12-month mark was executed via structured telephone interviews focusing on patient-reported outcomes and symptomatic recurrence screening.

### Measures of outcome

*Primary*:

- Postoperative chronic pain, defined as pain that has newly developed after surgery, has increased in intensity, or has different characteristics from pre-existing pain, that persists for at least 3 months following a procedure, lasting beyond the expected healing time, and is not caused by other factors like infection [32]. It was verified at 3 months and 6 months postoperatively, and reported in number of cases /total.

*Secondary*:

- Clinical Safety (Time frame for verification:1 day, 1 or 2 weeks, 1 month, 3 months, 6 months)
- Intraoperative blood loss (in ml)
- Rate of postoperative complications. The Clavien-Dindo Scale was used to standardize the severity of perioperative complication across different hospitals [33]. These include:
  - wound infection (n. events/total number of patients)
  - local hematoma formation (n. events/total number of patients)
  - seroma persisting beyond 90 days (n. events/total number of patients)
  - re-hospitalization for severe complications (Clavien-Dindo IV, in events/total).
- early postoperative pain (24 h from the hernia procedure), as measured on a visual analog scale (VAS) from 0–10
- pain at hospital discharge as measured on a VAS from 0–10
- Efficiency:
- Operating time (in minutes), defined as time interval from incision to final skin closure.
- Postoperative duration of stay (in days).
- Effectiveness [Time Frame for postoperative verification: 1 day, 1 or 2 weeks, 3 months, 6 months, 12 months]
- Verification of hernia recurrence in cases / total. A clinical examination was conducted to evaluate recurrence. If there was any suspicion of recurrence, an ultrasound, MRI or CT is performed according to hospital practice to distinguish true hernia recurrences from bulging. Technical success of glue fastening was be evaluated intra-operatively, the investigator registered whether mesh fixation system achieved technical success or not. Technical success is defined as successful hernia repair as intended.

### Sample size calculation

The primary objective of this study was to evaluate the impact of non-penetrative versus penetrative mesh fixation on the incidence of chronic postoperative pain at 6 months. Based on current literature, the expected incidence of chronic pain defined as a binary outcome (presence vs. absence of bothersome or clinically significant pain at 6 months [34]) is 20% in the penetrative fixation group (Control) [35, 36].

We hypothesized that non-penetrative fixation (Experimental) will reduce this incidence to 7%.

To detect this absolute risk reduction of 13% with a two-sided alpha (α) of 0.05 and a power (1 − β) of 0.80, a sample size of 96 patients per arm is required. A target number of 100 patients per arm was established, accounting for potential attrition during follow-up and occasional incomplete data, to maintain adequate sample size for statistical significance.

### Statistical analysis

Statistical analysis was performed on an Intention-to-Treat (ITT) basis. The evaluation of outcomes stratified by surgical approach (open vs. laparoscopic) was pre-specified within the prospective data analysis plan prior to statistical evaluation. Baseline demographic and clinical characteristics (e.g., BMI, hernia defect size, primary vs. incisional hernia) are summarized using descriptive statistics. Continuous variables are expressed as mean ± standard deviation (SD), and categorical variables as frequencies and percentages.

Univariate analysis was conducted on:

- *Primary endpoint*: The proportion of patients reporting chronic pain at 6 months, compared using the Chi-square test or Fisher’s exact test as appropriate.
- *Secondary endpoint (efficiency)*: Mean operating time (minutes) was compared using an Independent Samples t-test. The study is powered to detect a small-to-moderate effect size (Cohen’s d = 0.40), which corresponds to a 3-minute difference in operative time assuming a standard deviation of 7.5 minutes.
- *Secondary endpoint (safety)*: intraoperative blood loss (in ml).
- *Secondary endpoint (clinical)*: VAS pain scores of the study and control group (in a scale 0-10), on first postoperative day and at hospital discharge respectively, were analyzed as continuous/ordinal variables. Group comparisons were performed using ANOVA (or non-parametric equivalents, such as the Mann-Whitney U test) on testing for normality (Shapiro-Wilk test), to ensure statistical validity when comparing mean or median VAS scores between the experimental and control groups at predefined postoperative intervals.
•*Secondary endpoint (clinical)*: Recurrence rates at 12 months was analyzed using Kaplan-Meier survival curves and compared via the Log-rank test.

Multivariable logistic regression analysis was conducted to identify independent predictors of chronic pain, adjusting for potential confounders such as surgical approach (open vs. laparoscopic), hernia type (primary vs. incisional), mesh type (synthetic, biosynthetic, composite), type of repair (onlay, sublay, intraperitoneal), defect size, comorbidities. For outcomes expressed by continuous data, multivariable linear regression was conducted to test all significant and clinically meaningful variable associations. All p-values are two-sided, with statistical significance set at *p*< 0.05. All analyses were conducted using the Statistical Package for Social Sciences (SPSS), ver. 26.0.

### Baseline imbalance & subgroup analysis

Baseline Characteristics and Covariate Adjustment. Continuous variables are summarized as means (SD) or medians (IQR), and categorical variables as frequencies (%).

Subgroup Analysis. The primary outcome is analysed in the total population and across the pre-specified strata of surgical approach (Laparoscopic vs. Open). We performed a test for interaction to determine if the effect of mesh fixation (cyanoacrylate vs. suture/tacks) is modified by the surgical approach. Both subgroup results are reported maintaining the integrity of the Intention-to-Treat (ITT) principle**.**

## Results

**Fig. 1:**
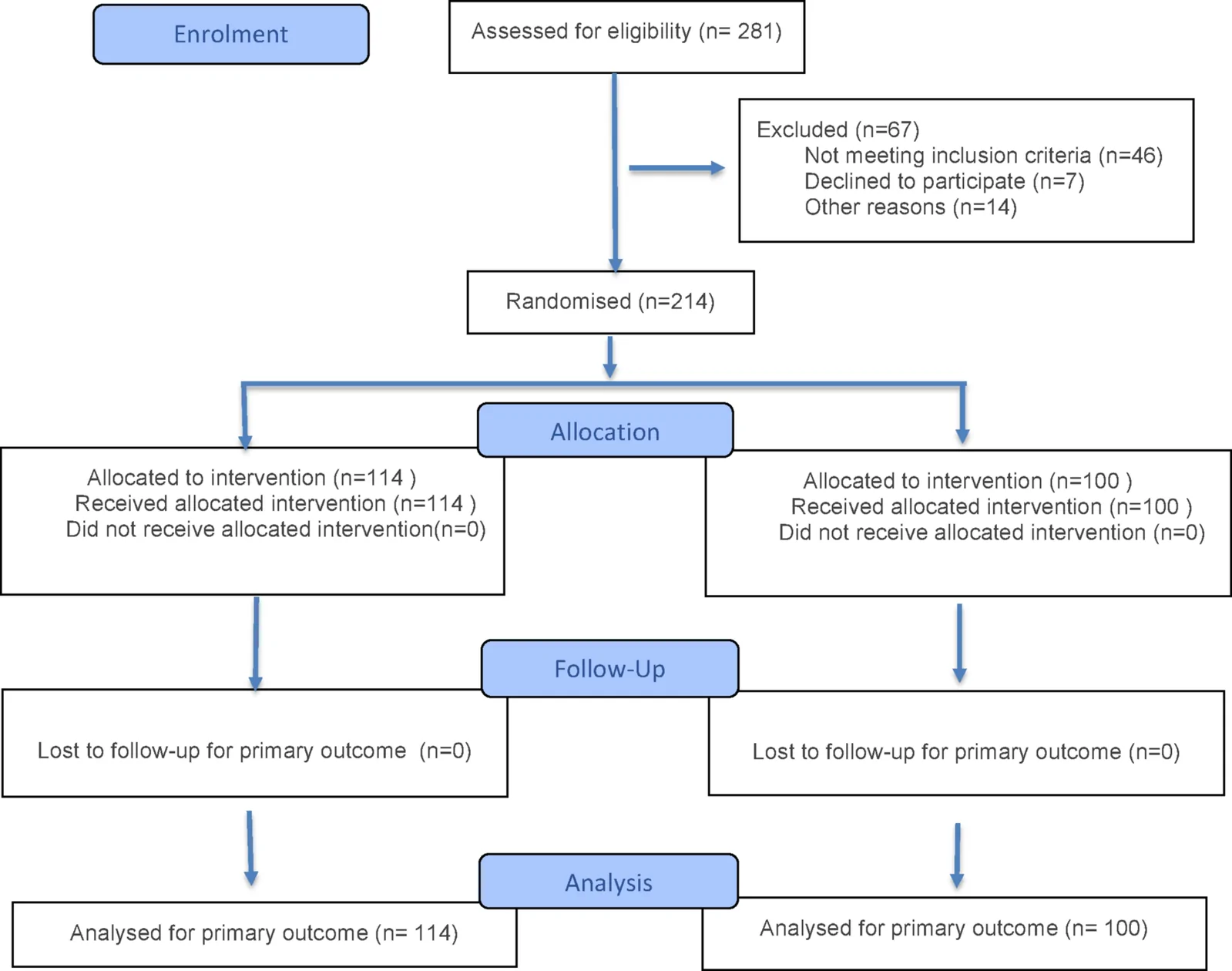
CONSORT 2025 flow diagram. Flow diagram of the progress through the phases of a randomised trial of two groups (that is, enrolment, intervention allocation, follow-up, and data analysis)

From March 2023 to February 2025, 281 patients with symptomatic, primary, or first-time incisional ventral hernias—regardless of hernia size—were referred to the participating centres and assessed for inclusion. Two hundred fourteen patients were enrolled to this study. All enrolled patients received the allocated treatment and were followed up according to protocol (Fig. 1).

The demographic and procedure characteristics of the study and control group are presented in Table 2.

**Table 2:** Demographic and procedure data of 214 patients undergoing ventral hernia repair with mesh

Patients		All patients	N (%), Mean (± SD)		P*
			Glubran	Transfixing fixation	
		214	114	100	
Age (years)		61.3 ± 13.3	61.6 ± 13.8	61.1 ± 12.9	0.753
Gender M (%)		115 (53.7)	63 (55.3)	52 (52)	0.681
Comorbidities					
	BMI	28.6 ± 3.4	28.3 ± 3.7	28.9 ± 3	0.310
	Smoker	54 (40)	33 (39.3)	21 (41.2)	0.858
	Charlson comorbidity Index	2.37 ± 1.6	2.46 ± 1.7	2.26 ± 1.5	0.358
ASA	I	9 (4.2)	5 (4.4)	4 (4)	0.410
	II	149 (69.6)	75 (65.8)	74 (74)	
	III	56 (26.2)	34 (29.8)	22 (22)	
Incisional		139 (65)	74 (64.9)	65 (65)	0.552
Defect size		4.95 ± 2.7	5.21 ± 3.1	4.66 ± 2.2	0.139
EHS size class	W1	102 (47.7)	54 (47.4)	48 (48)	0.994
	W2	95 (44.4)	51 (44.7)	44 (44)	
	W3	17 (7.9)	9 (7.9)	8 (8)	
Approach	Open	105 (49.1)	61 (53.5)	44 (44)	0.165
	Minimally invasive	109 (50.9)	53 (46.5)	56 (56)	
Component separation		5 (2.3)	4 (3.5)	1 (1)	0.225
Repair type^*^	Onlay	5 (2.3)	3 (2.6)	2 (2)	0.417
	Sublay	108 (50.5)	62 (54.4)	46 (46)	
	IPOM	101 (47.2)	49 (43)	52 (52)	
Mesh type	Synthetic	55 (25.7)	34 (29.8)	21 (21)	0.323
	Biosynthetic	11 (5.1)	6 (5.3)	5 (5)	
	Composite	148 (69.2)	74 (64.9)	74 (74)	
Mean Follow-up (months)		24.6 ± 7.5	24.2 ± 7.4	24.9 ± 7.6	0.624

SD—standard deviation; BMI—body mass index; ASA—American Society of Anaesthesiology; COPD—Chronic obstructive pulmonary disease; *In the control group, slow-absorbing monofilament/barbed sutures were predominant in open surgery

The most frequently performed repair techniques were sublay and intraperitoneal mesh repair. Less than one tenth of the study groups were composed by hernias with a defect 10 cm and above.

At the 6-month assessment, the overall incidence of chronic pain was 5.6% (12/214 patients), with no significant differences between the two groups (Table 3). All cases of chronic pain occurred exclusively within the open surgery cohort (12/105, 11.4%), whereas no cases of chronic pain (0/109, 0%) were recorded in the laparoscopic cohort.

**Table 3:** Perioperative outcomes of 214 patients undergoing ventral hernia repair with mesh

Patients			All patients	N (%), Mean (± SD)		P*
				Glubran	Transfixing fixation	
			214	114	100	
Operating time (min)			80.8 ± 40.6	75.6 ± 38.3	86.8 ± 42.4	0.043
Intraoperative blood loss (ml)			24.5 ± 26.6	24.2 ± 28.3	24.9 ± 24	0.866
Postoperative stay (days)			4 ± 4.1	3.8 ± 4.6	4.3 ± 3.5	0.431
Drain removal interval time (days)			2.1 ± 2.8	2.3 ± 2.4	1.8 ± 2.8	0.117
Postoperative complications	Overall		67 (31.3)	30 (26.3)	40 (40)	0.033
	Clavien Dindo class	0	147 (68.7)	84 (73.7)	63 (63)	0.394
		1	55 (25.7)	24 (21.1)	31 (31)	
		2	10 (4.7)	5 (4.4)	5 (5)	
		3b	2 (0.9)	1 (0.9)	1(1)	
	Seroma		29 (13.6)	13 (11.4)	16 (16)	0.327
	Hematoma		14 (6.5)	8 (7)	6 (6)	0.764
	Infection		6 (2.8)	2 (1.8)	4 (4)	0.467
	Mesh rejection		1 (0.5)	0 (0)	1 (1)	0.467
	Other complications		8 (3.7)	5 (4.4)	3 (3)	0.726
	Reintervention		4 (1.9)	3 (2.6)	1 (1)	0.379
Ist PO day pain (VAS scale)			3.8 ± 1.3	3.7 ± 1.3	3.9 ± 1.2	0.337
Discharge day pain (VAS scale)			1.89 ± 1.2	1.7 ± 2.5	2.11 ± 1.2	0.017
Chronic pain			12 (5.6)	6 (5.3)	6 (6)	0.815
Recurrence at 12 months			1 (0.4)	1 (0.8)	0	0.500

SD—standard deviation; BMI—body mass index; ASA—American Society of Anaesthesiology; COPD—Chronic obstructive pulmonary disease; *significance for Chi-square test is 2-sided

In the study group, overall perioperative complications and postoperative pain at hospital discharge were also respectively significantly less frequent and of lower intensity. Operating time was significantly shorter in procedures where hernia mesh was fixated with cyanoacrylate (Table 3).

Overall, the occurrence of postoperative hematoma was the only independent factor increasing the odds of postoperative chronic pain at multivariable analysis (OR 5.036, (95% CI: 1.042–23.349, *p* = 0.044—Table 4).

**Table 4:** Multivariable analysis of perioperative outcomes (All cases)

	Postoperative Chronic pain		Overall perioperative complications		Operating time		Intraoperative blood loss		Pain at hospital discharge	
Factor	Exp (B) (95%CI)	*p*	Exp (B) (95%CI)	*p*	Unstandardized B (95%CI)	*p*	Unstandardized B (95%CI)	*p*	Unstandardized B (95%CI)	*p*
Cyanoacrylate versus transfixing mesh positioning	–		0.457 (0.244–0.855)	**0.014**	− 7.172 (− 23.040/− 2.008)	**0.020**	–		− 0.356 (− 0.691/− 0.022)	**0.037**
Incisional versus primary ventral hernia	–		–		6.445 (5.517–30.929)	**0.005**	–		–	
Laparoscopic approach	–		–		–		− 21.357 (− 33.246/− 9.468)	**0.001**	–	
Postoperative Hematoma	5.036 (1.042–23.349)	**0.044**	–		–		–		–	

Subgroup analysis showed nonsignificant differences in the demographic data and outcomes of patients treated by open approach (Appendix Table 8). At 12 months, there was one recurrence in the open VHR subgroup.

In the laparoscopic stratum, there were no significant differences in the demographic characteristics of the experimental and control group (Table 5).

**Table 5:** Demographic data of 109 patients undergoing minimally invasive ventral hernia repair with mesh

Patients		All patients	N (%), Mean (± SD)		P*
			Glubran	Transfixing fixation	
		109	53	56	
Age (years)		58.8 ± 13.6	56.2 ± 12.1	61.3 ± 14.6	0.054
Gender M (%)		61 (56)	31 (58.5)	30 (53.6)	0.700
Comorbidities					
	BMI	28 ± 3.8	27.7 ± 3.4	28.2 ± 4	0.580
	Smoker	15 (45.5)	10 (43.5)	5 (50)	0.730
	Charlson comorbidity Index	1.7 ± 1.2	1.4 ± 1	1.9 ± 1.4	0.077
ASA	I	3 (2.8)	2 (3.8)	1 (1.8)	0.792
	II	82 (75.2)	40 (75.5)	42 (75)	
	III	24 (22)	11 (20.8)	13 (23.2)	
Incisional		45 (41.3)	17 (32.1)	28 (50)	0.080
Defect size		4.2 ± 2	4.1 ± 2	4.3 ± 2	0.513
EHS size class	W1	63 (57.8)	35 (66)	28 (50)	0.200
	W2	42 (38.5)	17 (32.1)	25 (44.6)	
	W3	4 (3.7)	1 (1.9)	3 (5.4)	
Component separation		0 (0)	0 (0)	0 (0)	n/a
Repair type^**^	Sublay	15 (13.8)	10 (18.9)	5 (8.9)	0.110
	IPOM	94 (86.2)	43 (81.1)	51 (91.1)	
Mesh type	Synthetic	16 (14.7)	11 (20.8)	5 (8.9)	0.200
	Biosynthetic	3 (2.8)	1 (1.9)	2 (3.6)	
	Composite	90 (82.6)	41 (77.4)	49 (87.5)	

SD—standard deviation; BMI—body mass index; ASA—American Society of Anaesthesiology; COPD—Chronic obstructive pulmonary disease; * significance for Chi-square test is 2-sided. There were no blood transfusions ^**^ absorbable, self-retaining fastener devices/tacks were predominant in laparoscopic IPOM; sublay approach consiste of an enhanced Transversus Abdominis Extraperitoneal (eTEP) approach—For the eTEP cases in the experimental group, liquid cyanoacrylate was applied strictly to the corners of the mesh to prevent migration in the retromuscular space; in the control group, slow-absorbing monofilament sutures were used. Every fixation was tracked transparently

No occurrence of chronic pain was recorded in the laparoscopic group for the duration of the trial. There was no recurrence at 12 months follow-up. Patients whose mesh was fixated with cyanoacrylate showed significantly less intraoperative overall complications, and in this group no complication occurred above Clavien-Dindo grade I (Table 6).

**Table 6:** Perioperative outcomes of 109 patients undergoing minimally invasive ventral hernia repair with mesh

Patients			All patients	N (%), Mean (± SD)		P*
				Glubran	Transfixing fixation	
			109	53	56	
Operating time (min)			71.8 ± 36.6	55 ± 28.2	87.7 ± 36.7	**< 0.001**
Intraoperative blood loss (ml)			10.4 ± 13	5.4 ± 8.8	19.4 ± 14.3	**< 0.001**
Postoperative stay (days)			3.7 ± 4.7	3 ± 5.2	4.4 ± 4	0.126
Drain removal interval time (days)			0.4 ± 1.2	0.3 ± 1.2	0.4 ± 1.2	0.584
Postoperative complications	Overall		24 (22)	5 (9.4)	19 (33.9)	**0.002**
	Clavien Dindo class	0	87 (79.8)	48 (90.6)	39 (69.6)	**0.038**
		1	18 (16.5)	5 (9.4)	13 (23.2)	
		2	3 (2.8)	0 (0)	3 (5.4)	
		3b	1 (0.9)	0 (0)	1 (1.8)	
	Seroma		16 (14.7)	4 (7.5)	12 (21.4)	**0.041**
	Hematoma		2 (1.8)	0 (0)	2 (3.6)	0.165
	Infection		1 (0.9)	0 (0)	1 (1.8)	0.328
	Mesh rejection		0 (0)	0 (0)	0 (0)	n/a
	Other complications		2 (1.8)	0 (0)	2 (3.6)	0.165
	Reintervention		1 (0.9)	0 (0)	1 (1.8)	0.328
Ist PO day pain (VAS scale)			3.7 ± 1	3.4 ± 0.8	3.9 ± 1.1	**0.005**
Discharge day pain (VAS scale)			2.0 ± 1.3	1.7 ± 1.4	2.3 ± 1.4	**0.039**
Chronic pain			0 (0)	0 (0)	0 (0)	n/a
Recurrence at 12 months			0 (0)	0 (0)	0 (0)	n/a

SD—standard deviation; BMI—body mass index; ASA—American Society of Anaesthesiology; COPD—Chronic obstructive pulmonary disease; * significance for Chi-square test is 2-sided. There were no blood transfusions

Also, use of Glubran 2 associated to a significantly lower postoperative time, intraoperative blood loss, postoperative stay and postoperative pain both on the first postoperative day and at discharge. At multivariale analysis, fixation with cyanoacrylate was independent factor for less overall postoperative complications and intraoperative blood loss, with the odds of one spared complication every 4–5 mesh fixation with cyanoacrylate and blood loss decrease of about 12 ml for each case of mesh fixation with cyanoacrylate (Table 7).

**Table 7:** Multivariable analysis of perioperative outcomes (laparoscopic approach)

	Overall perioperative complications		Operating time		Intraoperative blood loss		Pain at hospital discharge	
Factor	Exp (B) (95%CI)	*p*	Unstandardized B (95%CI)	*p*	Unstandardized B (95%CI)	*p*	Unstandardized B (95%CI)	*p*
Cyanoacrylate versus transfixing mesh positioning	0.235 (0.078–0.715)	**0.011**	− 31.800 (− 41.316/− 22.284)	**< 0.001**	− 12.305 (− 20.883/− 3.726)	**0.021**	− 0.535 (− 1.024/− 0.047)	**0.032**
Incisional versus primary ventral hernia	–		–		–		0.590	
(0.095–1.084)	**0.020**							
Defect size in cm	–		9.797 (7.406–12.128)	**< 0.001**	–		–	
Ipom versus Sublay repair	–		− 16.682 (− 30.741/− 2.623)	**0.020**	–		− 1.108 (− 1.809/− 0.406)	**0.002**

Non-transfixing positioning was also a factor for significantly decreased operating time, though procedure time was also function of the defect size and choice of repair in this study. Pain at hospital discharge was reduced by fixation method and IPOM repair, while it was significantly increased in incisional hernia cases (Appendix Tables 8, 9).

No statistically significant interaction was detected between fixation method and surgical approach.

## Discussion

This multicentric, pragmatic randomized trial evaluated the clinical outcomes of non-transfixing mesh fixation using a synthetic cyanoacrylate-based glue (Glubran® 2) versus mechanical transfixion (sutures or tacks) in VHR. By incorporating both open and laparoscopic approaches, the study aimed to address the fundamental clinical trade-off in herniology: the maintenance of prosthetic stability versus the minimization of mechanical tissue trauma.

### Chronic pain and fixation methodology

In this cohort, all chronic pain events occurred after open repair, suggesting that factors related to surgical access and tissue dissection may have contributed more substantially than fixation method itself. This clinical reality is supported by multivariable analysis, which identified postoperative hematoma as the sole independent predictor of chronic pain, increasing the risk fivefold.

The primary endpoint of this study—chronic postoperative pain at six months—did not differ significantly between the study and control groups, with an overall incidence of 5.6%. This finding suggests that, in a real-world surgical setting, the specific method of mesh anchoring may not be the primary determinant of long-term discomfort. These results align with a similar randomized trial by Bhattacharjee et al. [37], which found no superiority for adhesive fixation regarding long-term pain, and also reflect findings from the Danish Ventral Hernia Database, where pain levels were comparable regardless of the anchoring device [38].

The reported incidence of “bothersome” chronic discomfort in the literature varies from 2 to 28% [31, 34]. The relatively low rate (5.6%) across both cohorts may be attributed to the participation of specialized centres and the standardization of surgical techniques. While it has been traditionally assumed that penetrative fixation causes nerve entrapment or fascial irritation, our data suggest that pain may instead be a function of general surgical trauma, the inflammatory response to the prosthetic material, or the extent of adhesiolysis [20, 21].

Notably, our multivariable analysis identified postoperative hematoma as the only independent predictor of chronic pain, increasing the odds fivefold.

While cyanoacrylate was an independent factor for reduced overall complications, it did not show a significant relationship with hematoma formation. This suggests that the relationship between fixation trauma and chronic pain is complex and likely impacted by secondary postoperative complications rather than the mechanical penetration of the abdominal wall alone.

### Perioperative efficiency and secondary outcomes

Significant differences were observed in acute perioperative outcomes. Operating time was significantly lower in the cyanoacrylate group, likely due to the rapid exothermic polymerization of the monomer, which bonds tissue in 4–5 s and reaches completion within 90 s. This efficiency was consistent across the cohort, though procedure time remained a function of defect size and the specific repair technique employed.

In the study group, pain intensity at hospital discharge was significantly lower than in the control group. This indicates that while “chronic” pain scores eventually converge due to biological healing and mesh integration, the immediate postoperative phase is better tolerated when atraumatic fixation is utilized.

### Subgroup analysis: laparoscopic versus open approach

In the laparoscopic stratum, the benefits of cyanoacrylate fixation were particularly pronounced. The use of Glubran® 2 was associated with significantly lower intraoperative blood loss (average decrease of 12 ml per case), reduced postoperative stay and complication rates, with no events exceeding Clavien-Dindo Grade I in the experimental group, and no recorded instances of chronic pain within the laparoscopic cohort.

These results suggest that the atraumatic nature of glue may be particularly advantageous during intraperitoneal onlay mesh (IPOM) repairs, where avoiding mechanical tacks may reduce the risk of visceral adhesions or injury to the peritoneum and underlying musculature [27, 28].

### Stability and recurrence rates

A critical concern regarding non-penetrating fixation is the risk of mesh migration and subsequent recurrence. Our study found no significant difference in recurrence rates at the 12-month follow-up, with only one recurrence in each arm of the open VHR subgroup and none in the laparoscopic group. These findings are consistent with experimental data showing that *N*-butyl 2-cyanoacrylate (NBCA) provides substantial tensile strength (34.27 N), ensuring adequate mesh integration [28, 30]. While some fibrin-based sealants have been associated with higher recurrence in humans [27], the synthetic cyanoacrylate used here appears to provide sufficient mechanical stability.

### Strengths and limitations

The primary strength of this study is its randomized, multicentric design and high follow-up rate (> 80%), which exceeds the threshold required for valid conclusions in clinical trials [39]. However, the pragmatic nature of the study—including primary and incisional hernias, as well as varied repair techniques (sublay, onlay, and IPOM)—introduced heterogeneity.

Several other limitations must be acknowledged when interpreting these results.

First, the overall incidence of chronic postoperative pain across the entire trial cohort was unexpectedly low. While clinically encouraging, this low event rate substantially underpowered our statistical analysis for the primary endpoint. As a result, our findings regarding chronic pain must be interpreted as exploratory rather than definitive. Also, because of the low number of chronic pain events, multivariable analyses were performed for exploratory purposes and may be susceptible to overfitting.

Second, patient follow-up at the 12-month mark was conducted primarily via telephone interviews rather than standardized, in-person clinical examinations. While telephone follow-ups are a practical and widely accepted method for long-term tracking in multicentric setups, they introduce a risk of underreporting subtle physical findings, such as subclinical recurrences or mild, intermittent discomfort that an in-person physical examination might otherwise detect.

Finally, while granular data on laparoscopic sub-techniques and surgeons' specific baseline experience were not captured, these procedural parameters represent performance and selection biases inherent to pragmatic, real-world data collection.

## Conclusion

This multicentric pragmatic trial demonstrates that cyanoacrylate mesh appears to be a safe and feasible alternative to traditional mechanical fixation methods in everyday clinical practice, displaying comparable perioperative outcomes with clinical advantages in terms of reduced operating time, decreased intraoperative blood loss, and improved acute pain management. However, due to the intrinsic confounding variations driven by surgical access, the independent impact of fixation methods on chronic postoperative pain cannot be universally declared. These findings should not be used to draw definitive conclusions across all techniques, but should instead serve as an empirical foundation. Further adequately powered, approach-specific randomized trials are warranted.

## Data Availability

Original population and Patients’ data supporting this study are available upon request from the Department of Precision and Regenerative Medicine and Jonian Area (DiMePRe-J), Academic General Surgery Unit “F. Paccione”.

## Appendix

**Table 8:** Demographic data of 105 patients undergoing open ventral hernia repair with mesh

Patients		All patients	N (%), Mean (± SD)		*P**
			Glubran	Transfixing fixation	
		105	61	44	
Age (years)					
Gender M (%)		54 (51.4)	32 (52.5)	22 (50)	0.480
Comorbidities					
	BMI				
	Smoker	39 (38.2)	23 (37.7)	16 (39)	0.528
	Charlson comorbidity Index				
ASA	I	6 (5.7)	3 (4.9)	3 (6.8)	0.165
	II	67 (63.8)	35 (57.4)	32 (72.7)	
	III	32 (30.5)	23 (37.7)	9 (20.5)	
Incisional		94 (89.5)	57 (93.4)	37 (84.1)	0.112
Defect size					
EHS size class	W1	36 (34.3)	16 (26.2)	20 (45.5)	0.122
	W2	57 (54.3)	37 (60.7)	20 (45.5)	
	W3	12 (11.4)	8 (13.1)	4 (9.1)	
Component separation		5 (4.8)	4 (6.6)	1 (2.3)	0.299
Repair type	Onlay	5 (4.8)	3 (4.9)	2 (4.5)	0.609
	Sublay	94 (89.5)	53 (86.9)	41 (93.2)	
	IPOM	6 (5.8)	5 (8.2)	1 (2.3)	
Mesh type	Synthetic	47 (44.8)	31 (50.8)	16 (36.4)	0.272
	Biosynthetic	8 (7.6)	5 (8.2)	3 (6.8)	
	Composite	50 (47.6)	25 (41)	25 (56.8)	

**Table 9:** Perioperative outcomes of 105 patients undergoing open ventral hernia repair with mesh

Patients		All patients	N (%), Mean (± SD)		P*
			Glubran	Transfixing fixation	
		105	61	44	
Operating time (min)		90.2 ± 42.5	93.4 ± 37.1	85.8 ± 49.3	0.366
Intraoperative blood loss (ml)		30.4 ± 28.7	32.8 ± 30	27 ± 26.6	0.311
Postoperative stay (days)		4.3 ± 3.4	4.5 ± 3.8	4.1 ± 2.7	0.546
Drain removal interval time (days)		3.9 ± 2.9	4.2 ± 2.6	3.5 ± 3.3	0.236
Postoperative complications	Overall	46 (43.8)	25 (41)	21 (47.7)	0.313
	Clavien Dindo class	60 (57.1)	36 (59)	24 (54.5)	0.570
		37 (35.2)	19 (31.1)	18 (40.9)	
		7 (6.7)	5 (8.2)	2 (4.5)	
		1 (1)	1 (1.6)	0 (0)	
	Seroma	13 (12.4)	9 (14.8)	4 (9.1)	0.288
	Hematoma	12 (11.4)	8 (13.1)	4 (9.1)	0.377
	Infection	4 (3.8)	2 (3.3)	2 (4.5)	0.466
	Mesh rejection	0 (0)	0 (0)	0 (0)	-
	Other complications	6 (5.7)	5 (8.2)	1 (2.3)	0.197
	Reintervention	2 (1.9)	2 (3.3)	0 (0)	0.335
Ist PO day pain (VAS scale)		4 ± 1.4	4 ± 1.5	3.9 ± 1.3	0.533
Discharge day pain (VAS scale)		1.7 ± 1.1	1.7 ± 1	1.9 ± 1.2	0.306
Chronic pain		12 (11.4)	6 (9.8)	6 (13.6)	0.381
Recurrence at 12 months		1 (1)	1 (1.6)	0 (0)	0.581

The only recurrence happened in a patient with W3, 15 cm diameter midline hernia

Univariable analysis of primary outcome chronic pain, all cases

**Table:**

		All cases, N	Chronic pain N (%) 12 (100)	*P*	Hospital stay (days)		Ist Postop Day Pain (VAS 0–10)		Pain at discharge (VAS 0–10)	
Overall postoperative complications	Yes	70	10 (14.3)	**< 0.001**	5.6 ± 6.2	**< 0.001**	4.4 ± 1.2	**< 0.001**	2.2 ± 1.2	**0.016**
	No	144	2 (1.4)		3.3 ± 2.2		3.6 ± 1.2		1.7 ± 1.2	
Hematoma	Yes	14	5 (35.7)	**< 0.001**	5.9 ± 6	0.086	4.5 ± 1.1	**0.039**	2.3 ± 1.3	0.224
	No	200	7 (3.5)		3.9 ± 3.9		3.8 ± 1.3		1.9 ± 1.2	
Seroma	Yes	29	1 (3.4)	0.497	6.7 ± 8	**< 0.001**	4.7 ± 1.1	**< 0.001**	2.8 ± 1.2	**< 0.001**
	No	185	11 (5.9)		3.6 ± 3		3.7 ± 1.2		1.7 ± 1.2	
Infection	Yes	5	1 (20)	0.358	12.4 ± 13	**< 0.001**	4.2 ± 0.8	0.508	2 + /0.7	0.846
	No	209	11 (5.2)		3.8 ± 3.5		3.8 ± 1.2		1.9 ± 1.3	
Other perioperative complication	Yes	8	1 (12.5)	0.375	10.6 ± 7.3	**< 0.001**	4.3 ± 1.4	0.228	1.9 ± 1.5	0.985
	No	206	11 (5.3)		3.7 ± 3.6		3.8 ± 1.2		1.9 ± 1.2	
Pain at discharge (VAS 0–10)	Yes	12	2.6 ± 1.2	**0.048**					-	
	No	202	1.8 ± 1.2							

Univariable analysis of primary outcome chronic pain, laparoscopic approach

**Table:**

		All cases, N	Hospital stay (days)		Ist Postop Day Pain (VAS 0–10)		Pain at discharge (VAS 0–10)	
Overall postoperative complications	Yes	24	8 ± 8.4	**< 0.001**	4.3 ± 1.2	**0.001**	2.7 ± 1.4	**0.002**
	No	85	2.5 ± 1.5		3.5 ± 0.9		1.8 ± 1.2	
Hematoma	Yes	2	15 ± 14.1	**< 0.001**	5 ± 1.4	0.066	3.5 ± 2.1	0.118
	No	107	3.5 ± 4.2		3.6 ± 1		2 ± 1.3	
Seroma	Yes	16	7.9 ± 8.9	**< 0.001**	4.7 ± 1	**< 0.001**	3.1 ± 1	**< 0.001**
	No	93	3 ± 3.1		3.5 ± 0.9		1.8 ± 1.2	
Infection	Yes	1	25	**< 0.001**	4	0.755	2	0.984
	No	108	3.5 ± 4.2		3.7 ± 1		2 ± 1.3	
Other perioperative complication	Yes	2	12 ± 2.8	**0.011**	3	0.347	1	0.277
	No	107	3.6 ± 4.6		3.7 ± 1		2 ± 1.3	

Univariable analysis of primary and secondary outcomes, all cases

**Table:**

		All cases, N	Chronic pain N (%) 12 (100)	*P*	Complications N (%) 70 (100)	*P*	Operating time (min)	*P*	Intraoperative blood loss	*P*	Hospital stay (days)	*P*	Ist Postop Day Pain (VAS 0–10)	*P*	Pain at discharge (VAS 0–10)	*P*
Ventral hernia	Primary	77	0 (0)	**0.004**	11 (15.7)	**< 0.001**	65 ± 33.6	**< 0.001**	11.1 ± 20.1	**0.003**	3.7 ± 5.4	0.484	3.6 ± 1	**0.030**	1.7 ± 1.2	0.182
	Incisional	137	12 (8.8)		59 (84.3)		90 ± 41.5		27.6 ± 27		4.2 ± 3.4		4 ± 1.4		2 ± 1.2	
Type of mesh positioning																
	Onlay	57	1 (8.3)	**0.018**	2 (28.6)	0.110	64.29 ± 18.1	**0.004**	10 ± 11	**0.026**	3.5 ± 2.4	0.655	3.7 ± 0.5	0.132	1.7 ± 0.8	0.880
	Sublay	108	11 (10.6)		43 (41.3)		90.6 ± 41.7		28.2 ± 28.7		4.2 ± 3.6		4 ± 1.4		1.9 ± 1.2	
	Ipom	101	0 (0)		25 (24.2)		72.68 ± 38.6		19.4 ± 3.1		3.8 ± 4.8		3.8 ± 1		2 ± 1.2	
Approach	Laparoscopic	109	0 (0)	**< 0.001**	24 (22)	**0.001**	71.8 ± 36.6	**0.001**	10.4 ± 13	**< 0.001**	3.7 ± 4.7	0.321	3.7 ± 1	0.070	2 ± 1.3	0.107
	Open	105	12 (11.4)		46 (43.8)		90.2 ± 42.6		30.4 ± 28.7		4.3 ± 3.6		4 ± 1.4		1.8 ± 1.1	
Mesh fixation	Cyanoacrylate	114	6(5.3)	0.523	30 (26.3)	**0.024**	75..6 ± 38.3	**0.043**	24.2 ± 28.3	0.866	4 ± 4.6	0.908	3.7 ± 1.3	0.337	1.7 ± 1.3	**0.017**
	Transfixing	100	6 (6)		40 (40)		42.4 ± 4.2		24.9 ± 24		4 ± 3.6		4 ± 1.2		2.1 ± 1.2	

Univariable analysis of primary and secondary outcomes, laparoscopic approach

**Table:**

		All cases, N	Complications N (%) 24 (100)	*P*	Operating time (min)	*P*	Intraoperative blood loss	*P*	Hospital stay (days)	*P*	Ist Postop Day Pain (VAS 0–10)	*P*	Pain at discharge (VAS 0–10)	*P*
Ventral hernia	Primary	64	7 (10.9)	**0.001**	62.4 ± 29	**0.001**	3.5 ± 7	**0.004**	3.8 ± 5.8	0.849	3.6 ± 0.9	0.223	1.8 ± 1.2	**0.022**
	Incisional	45	17 (37.8)		85.2 ± 42.2		14.8 ± 14		3.6 ± 2.5		3.8 ± 1.2		2.4 ± 1.4	
Type of mesh positioning														
	Bridged IPOM	2	0 (0)	0.493	85 ± 21.2	**0.087**	10 ± 14.1	**0.465**	4 ± 1.4	0.983	4	0.667	2.5 ± 0.7	**0.045**
	Sublay	15	2 (13.3)		90.3 ± 31		6 ± 9.7		3.5 ± 5		3.9 ± 1.1		2.8 ± 1.3	
	Ipom	92	22 (23.9)		68.5 ± 37		11.9 ± 13.8		3.7 ± 5		3.6 ± 1		1.9 ± 1.3	
Mesh fixation	Cyanoacrylate	53	5 (9.4)	**0.002**	55 ± 28.2	**< 0.001**	5.4 ± 8.8	**< 0.001**	3 ± 5.3	0.126	3.4 ± 0.9	**0.005**	1.7 ± 1.4	**0.039**
	Transfixing	56	19 (33.9)		87.7 ± 36.7		19.4 ± 14.4		4.4 ± 4		4 ± 1.1		2.3 ± 1.2	

There are no cases of chronic pain, this variable is not computed
